# A survey of *laxoox/canjeero*, a traditional Somali flatbread: production styles

**DOI:** 10.1186/s42779-022-00138-3

**Published:** 2022-06-21

**Authors:** Erin Wolgamuth, Salwa Yusuf, Ali Hussein, Antonella Pasqualone

**Affiliations:** 1 Dubai, UAE; 2 Hargeisa, Somaliland; 3 Hodan District, Mogadishu, Somalia; 4 Department of Soil, Plant and Food Science (DISSPA), Food Science and Technology Unit, University of Bari ‘Aldo Moro’, Via Amendola 165/a, 70126 Bari, Italy; 5 Brussels Institute of Advanced Studies (BrIAS), Fellow 2021/22, Brussels, Belgium

**Keywords:** Somalia, Traditional food, Ethnic food, Food culture, Pancake-like flatbread, Sorghum, *Cajiin*, *Dhanaanis*, *Dhaawe* or *dawa*

## Abstract

Flatbreads are traditional food products of ancient origin, still produced and appreciated in their original locations and beyond, having spread through historical and modern migration. While the history and preparation of Somali flatbread, known as *canjeero* in southern regions and *laxoox* in northern regions, has been shared orally for generations among the women who cook it at home, it has not been the object of scientific studies. An in-field study was therefore carried out in various cities in Somalia, Somaliland, and Ethiopia’s Somali State to document the formulation, production methods, and consumption patterns of this Somali flatbread. *Laxoox*/*canjeero* production was found to be relatively homogenous, but the data revealed two significant divergences: in bread formulation and in the procedure for structure development. These divergences result from disparities in the mechanization of bread production between rural and urban, and from the destruction of infrastructure, including food processing machines in public markets, leading up to the Somali civil war. An original framework of four production styles (“heritage,” “new heritage,” “innovative,” and “global”) illustrates these divergences in detail. Heritage production is linked to the historic era of nomadic Somali pastoralism, while the other three styles originated in civil conflict and continue today.

**Graphical abstract:**

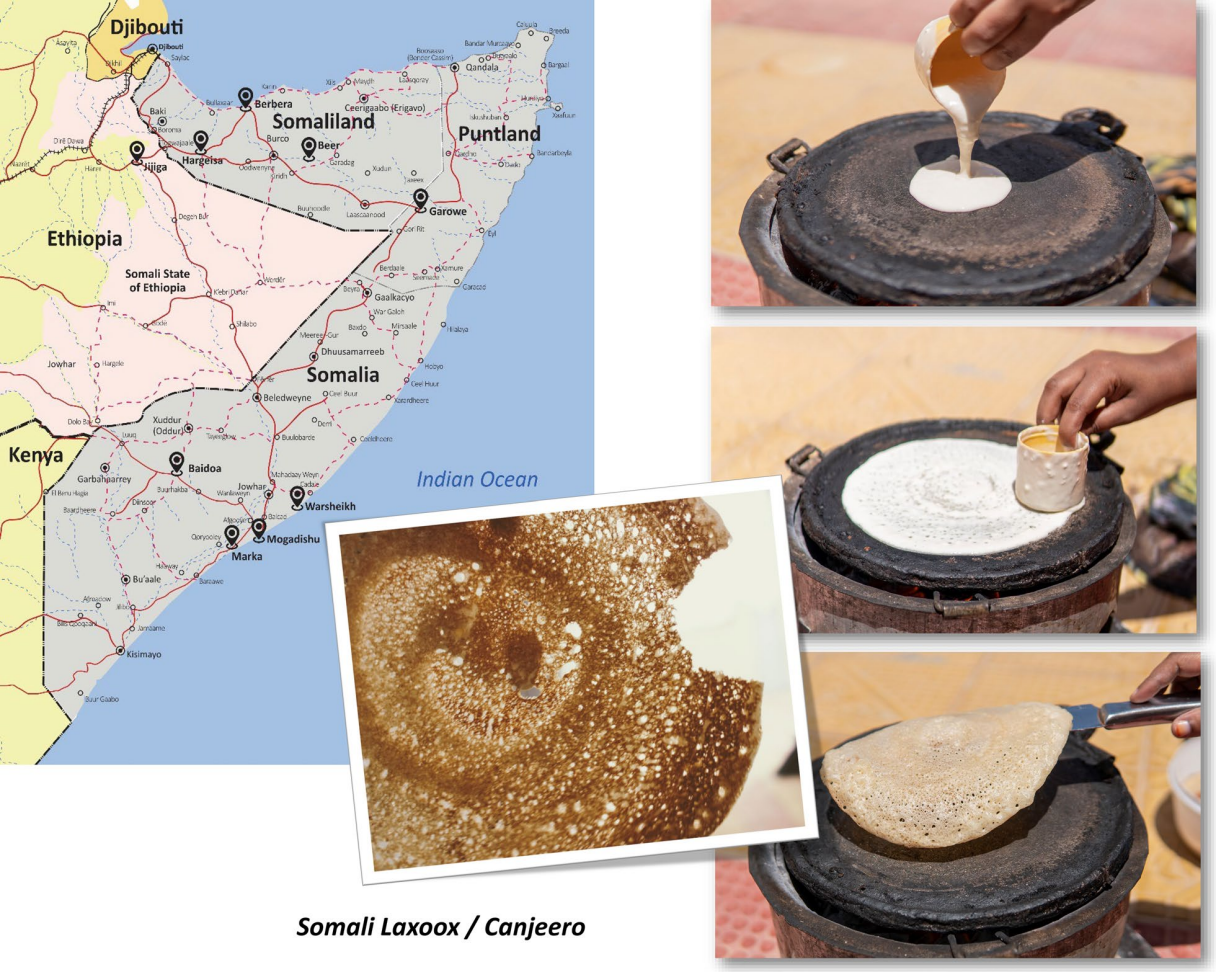

**Supplementary Information:**

The online version contains supplementary material available at 10.1186/s42779-022-00138-3.

## Introduction

The diversity of global breads is vast. Bread is among the foods most representative of identity and traditions and often carries religious or other symbolic meaning [1]. Bread types may correspond to specific countries, to subregions or ethnic groups, or cross-boundaries and borders through affiliation with multi-national or diaspora cultures. Bread is also valued for its association with specific rituals such as weddings [2] and funerals [3] or spiritual rites, such as breaking religious fasts. Bread has attracted the interest of scholars for more than a millennium. The ancient Greek scientist Oribasius (fourth–fifth century CE) collected an enormous body of information on different types of bread eaten in late antiquity and their dietary values [4]. In Persia, Bahāʾ al-Dīn Muḥammad ibn Husayn al- Āmilī (also known as Sheikh Baha’I, late sixteenth–early seventeenth centuries CE) [5] and Mohammad Hossein Aghili Khorasani Shirazi (seventeenth century CE) described the characteristics of various breads based on flour type, bran quantity, and baking method, as catalogued in the Persian medical book Makhzan al-Adviyeh [6]. Through the ages, bread has been an essential food product in nearly every culture.

In Somalia, while rice and pasta are contemporary staple foods (a result of historical trade and colonization), a diverse array of breads accompany stews, sauces, and other common dishes [7]. These include breads baked in large earthen ovens, such as *rodhi moofo* (literally “oven bread”), breads baked in vertical ovens, like the maize-based *muufo*, and breads borrowed directly from historical trade partners, such as *chapati* (from India via East African trade) and *malawah* (from Yemen). These breads are consumed with varying frequency or in specific locations (e.g., specific subregions, urban versus rural areas, and agricultural zones).

One bread, in contrast, is consumed daily in virtually all Somalia households in the Horn of Africa and by global Somali diaspora, most commonly for breakfast [7]. It is a fermented flatbread with a slightly sour flavor and spongy texture [7] known as “*laxoox*” (*la-HOH)* in the north (namely in Somaliland, a de facto independent country since 1991 that is unrecognized by the international community) [8] and as “*canjeero*” (*an-JER-o*) in southern Somalia. This bread stands apart from others for its ubiquity, frequency of consumption, and strong association with Somali identity and culture. One of the earliest written references to this ethnic bread may be found in the 1870s travel logs of British Lieutenant Captain Charles J. Cruttenden of the Indian Navy, stationed in Aden, Yemen, where Somalis had worked, traded, and lived for at least a century. Cruttenden noted that Somali women “go about vending cakes of fermented and unfermented bread” [9].

Globally, flatbreads are traditional food products with ancient origins that are still produced and appreciated today in their places of origin and beyond. Emerging from subsistence economies in multiple geographic areas (from the Mediterranean Basin to the Indian subcontinent, through the Horn of Africa and the Middle East), the various types of flatbreads can be categorized based on (1) the consistency of the starting mixture, ranging from a fluid batter to a visco-elastic dough; (2) the presence or absence of fermentation; (3) the baking system; and (4) the thickness and structure (single- or double-layered) [10]. Somali *laxoox*/*canjeero* fits the category of pancake-like flatbreads, i.e., those made from a batter comprised typically, though not exclusively, of legumes or of cereals other than wheat, usually due to a scarcity of wheat in production areas [10].

Bread is never simply food; it is linked to culture and personal experience, including childhood memories. In a relevant example, the sound of vigorous hand-mixing of the *laxoox/canjeero* batter, conventionally undertaken in the evening by women in the household, has been romanticized in Somali popular culture as the sound of nightfall: “Every night, before the cities and towns of Somalia go to sleep, you will hear rhythmic sounds of slapping, the sound of mixing the [*canjeero*] batter. It is a comforting and reassuring sound that lulls you to sleep, knowing that tomorrow’s meal is being prepared” [11]. *Laxoox/canjeero* is significant in its status as an essential element of the daily Somali diet and an inexpensive source of nutrition. Given its quotidian nature, *laxoox/canjeero* is not usually eaten on special occasions. It is, however, commonly eaten alongside special dishes at the sunset *iftar* meal to break the day’s fast during Ramadan, the holy Islamic month of fasting [12]. Like other ethnic foods, *laxoox*/*canjeero* is a tangible sign of Somali culture and carries value in light of recent decades of sociopolitical fracture, war, and population displacement that have led to a significant global diaspora.

Somali culture is traditionally oral; thus, very few written records exist on historical cookery. However, since at least the Adal Sultanate of the thirteenth century, pastoralists and agro-pastoralists have traversed the borders of present-day Somalia [13], including to and from Ethiopia, where there are many parallels to Somali foodways. For example, traditional cooking by nomadic Somalis was historically performed atop wood charcoal set over three stones in a ground pit (known in Somali as *dhardhaar*) [14], as it was in Ethiopia [15]. This style of cooking persisted in Somalia into the twentieth century, as recounted by elderly respondents. Ethiopian *injera*, a fermented flatbread considered by many to be a “cousin bread” of *laxoox/canjeero*, dates in written record to the fourteenth century CE, although the earliest evidence of the *mitad*, the griddle upon which Ethiopian *injera* is cooked, dates further back to the fifth or sixth century CE [16]. In another comparison, Yemeni *lahoh*, another regional fermented flatbread, dates in written record to the tenth century CE [17]. Given that agriculture (and resultant processing of cultivated grains and cereals) in Ethiopia and Yemen precedes the development of agriculture in Somalia, we presume that these years represent early boundaries for the appearance of Somali *laxoox/canjeero*.

While the history and preparation of Somali flatbread has been shared for generations among the women who commonly cook it at home, it has not thus far been the object of scientific studies. Over the last ten years (2012–2021), no results appear in the Scopus scientific database that include “*canjeero,*” “*laxoox,*” (or alternative spellings) or “Somali flatbread” in the article title, abstract, or keywords. A more generic search for “Somali bread” shows four documents which mention bread in wider contexts, without considering production technology, origins, or tradition [18]. To compare this with existing works on similar breads, Ethiopian *injera* has been the object of greater—and growing—attention by researchers, with 26 articles published in the period 2012–2016 showing “*injera*” in the title, abstract, or keywords, and 62 articles in the most recent 5-year period (2017–2021) [18]. Besides Ethiopian *injera* [19], studies have established the presence of similar traditional flatbreads in the broader region, including the *ambasha*, a fermented pancake-like bread, prepared by the Eastern Tigray people of Ethiopia and of Eritrea, both geographically proximate to Somalia [20]. Among the Eastern Tigray, non-fermented flatbreads such as *torosho* and its dry version *birkuta* have also been reported [20]. All of these breads have been recently investigated with the aim of reviewing both traditional ethnic foodways and scientific studies related to their preparation [19, 20].

Given the dearth of literature on Somali *laxoox/canjeero*, the aim of this article is to document, by means of an in-field study, the contemporary in situ composition, production methods, and consumption patterns of this flatbread, with historical reference where available. This article, which analyzes the rationale of the production steps with the help of the scientific knowledge of food science and technology, offers the following value addition to existing and relevant literature: (1) It is the first-ever scientific analysis of any Somali flatbread to be published in an academic journal; thus, it is unique; (2) it sheds light on previous studies of regional (e.g., Ethiopia, Eritrea, Yemen), continental (e.g., Sudan), and global (e.g., Baltic and western countries) breads through new comparisons of production steps, final products, and consumption; and (3) it incorporates sociopolitical and cultural dynamics to understand the drivers of change in the evolution of flatbread production, which can elucidate similar changes beyond Somali territories.

## Materials and methods

### Survey area

**Fig. 1:**
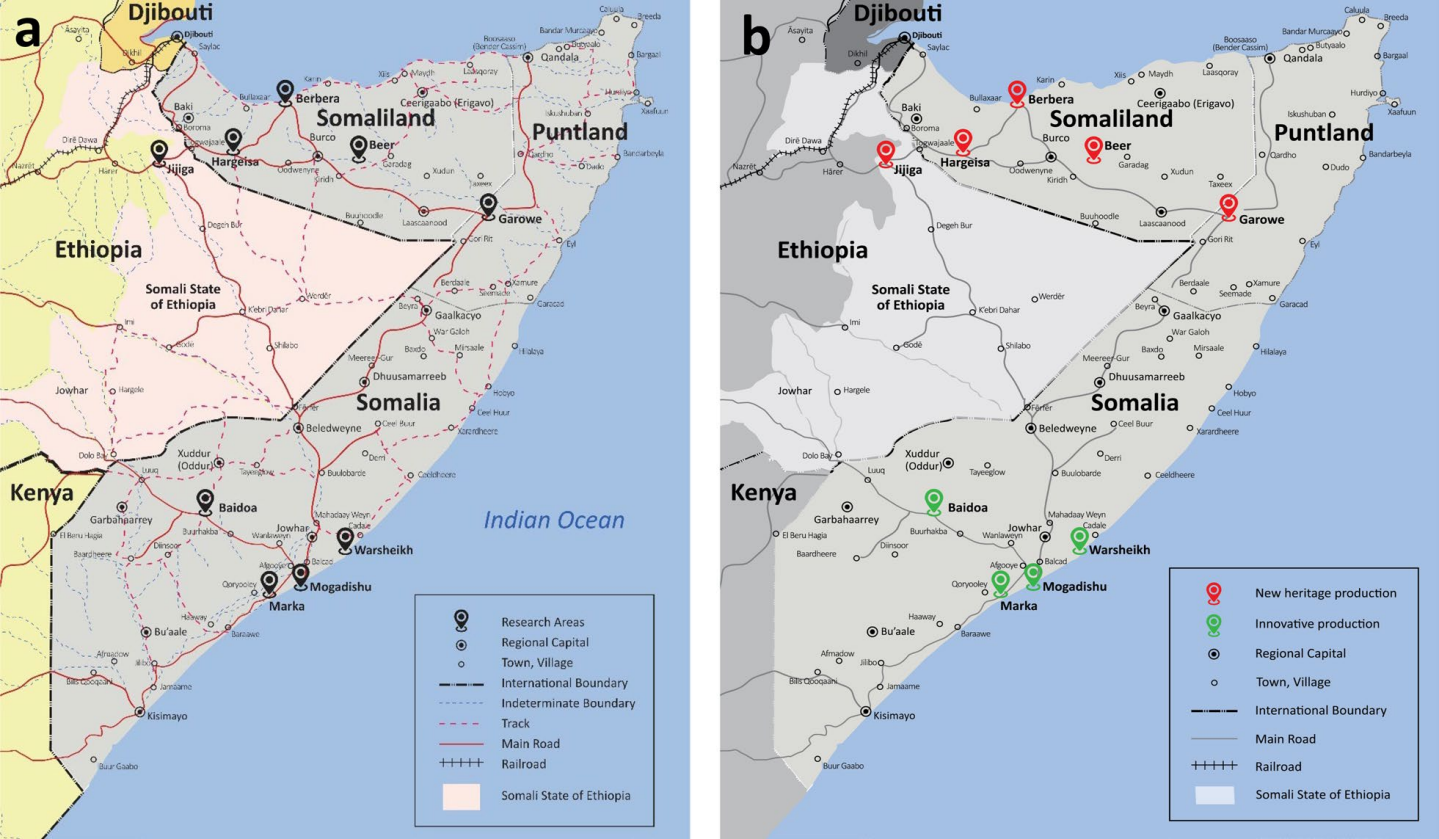
Map of research locations and *laxoox*/*canjeero* production styles. **a** The research locations included: (1) eight urban and peri-urban locations (in Somalia, Puntland, and Somaliland, the latter being a de facto independent country as-yet unrecognized by the international community); (2) one urban location in an ethnically Somali zone of Ethiopia, known as the Somali State. In detail, research locations were: Baidoa, Warsheikh, Mogadishu, and Marka (Somalia), Garowe (Puntland), Hargeisa, Berbera, and Beer (Somaliland), and Jigjiga (Ethiopia's Somali State). **b** Geographic areas indicated by “new heritage” and “innovative” production styles. New heritage production was found in Somaliland, in Ethiopia’s Somali State, and in rural areas of southern Somalia. Innovative production was found namely in urban southern and eastern Somalia

To account for varying bread-making traditions in different regions, the surveyed areas included: (1) four locations (Baidoa, Warsheikh, Mogadishu, and Marka) in Somalia; (2) one location (Garowe) in Puntland; (3) three locations (Hargeisa, Berbera, and Beer) in Somaliland; and (4) one location (Jigjiga) in an ethnically Somali zone of Ethiopia, known as the Ethiopia’s Somali State. Research locations are reported in Fig. 1.

### Data collection

Data on the production process of *laxoox*/*canjeero* were collected by means of direct, structured interviews based on a questionnaire (Additional file 1). Interviewees were asked to respond according to their personal knowledge and experiences, and ambiguities and partial answers were clarified through follow-up communications. Interviews were carried out in the local language (i.e., Somali with some minor variations to reflect differences in regional vocabulary) and then translated into English. Field researchers further documented household flatbread production via photographs and video in Hargeisa, Mogadishu, and Jigjiga. Data collection was completed in 7 months, from July 2020 to February 2021.

### Informants

Forty-six informants were involved in the study. All were adult Somali women who regularly prepare *laxoox/canjeero* for their households, ranging in age from 17 to 70 years (average age 34). Informants were selected via convenience sampling with the following constraints: Each had to be from a different household, and none could have ever shared a household. This was to avoid the selection of, for example, sisters who currently reside in separate households but were reared together and therefore whose bread production styles may coincide as a result of shared learning. All participants were informed about the purpose of the study, adherence to data confidentiality protocols, that their participation was voluntary, and that they could leave the interview at any time if they wished to do so. Formal consent was obtained before starting the interviews.

### Questionnaire on *laxoox/canjeero*

The questionnaire on *laxoox/canjeero* focused on bread formulation, batter preparation, cooking, and consumption patterns. It contained numerous and specific closed-ended questions, with the intention of understanding the food science rationale behind flatbread production and to make the entire process clear for readers unfamiliar with this kind of bread. The questionnaire was preliminarily tested with four people to ensure comprehension and was adapted to each survey location with the use of the appropriate local name of the flatbread, i.e., *canjeero* in Baidoa, Warsheikh, Mogadishu, Marka, and Garowe, and *laxoox* in Hargeisa, Berbera, Beer, and Jigjiga.

### Questionnaire on *cajiin*

Over the course of data collection, the use of a pre-gelatinized dough known locally as “*cajiin*” (from the Arabic “*al ajiin*”, meaning “dough”) was revealed to be common in flatbread production in urban southern Somalia (Baidoa, Warsheikh, Mogadishu, and Marka). To better understand the procedure for developing *cajiin*, as well as consumer preferences regarding its use, a separate questionnaire was developed, titled “Questionnaire on *cajiin*” (Additional file 2). The latter contained open-ended questions and was structured in two sections. The first section detailed the *cajiin* description and production process, as well as sale and usages beyond *laxoox/canjeero*. The second section covered more general questions about the introduction of *cajiin* in the Somali culinary repertoire and its advantages over alternative products used for similar purposes in household cooking. The questionnaire was administered to four experts: two *cajiin* producers (men, 32 and 35 years old) and two *cajiin* salespeople (women, 40 and 43 years old).

### Data analysis

All data were reviewed for accuracy and completeness, and responses from the questionnaires were entered into Excel (Microsoft Office Version 2018 for Windows, Microsoft Corporation, Redmond, Washington, USA) to analyze the frequency distribution of each answer.

## Results and discussion

**Fig. 2:**
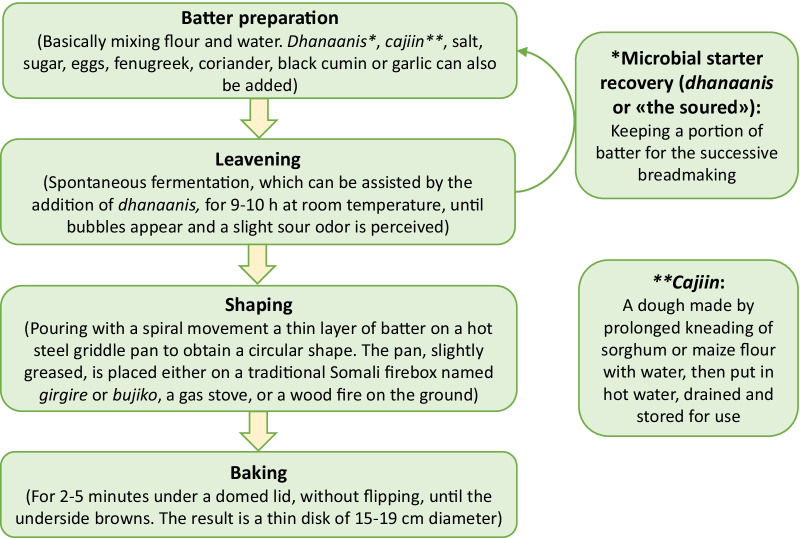
Production flowchart of *laxoox*/*canjeero* illustrating the operative conditions of each processing step: batter preparation, leavening, shaping, and baking, as well as *cajiin* production and *dhanaanis* recovery. *Laxoox*/*canjeero* fits the category of pancake-like flatbreads, i.e., those obtained by baking a batter comprised typically, though not exclusively, of legumes or from cereals other than wheat, usually due to a scarcity of wheat in production areas. *Cajiin* is a pre-gelatinized dough, while *dhanaanis* is a fermentation starter

**Fig. 3:**
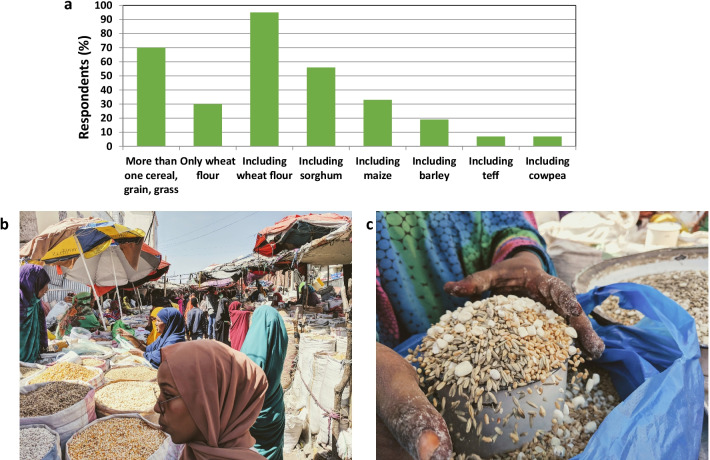
Type of flour used in the production of *laxoox*/*canjeero*. **a** The majority of respondents included wheat flour in the batter, followed by those who included sorghum, maize, barley, teff, and pulses such as cowpea and adzuki beans. Thirty percent of respondents used only wheat flour to prepare the batter; of this group, all were located in southern Somalia. In Somaliland and Ethiopia, by contrast, all respondents reported using at least two grains, cereals, or grasses in their flatbread mixtures. **b** A grain market in downtown Hargeisa, where female vendors sell individual grains, cereals, beans, and legumes, as well as unique mixtures tailored to common dishes. **c** A sample flatbread grain and cereal mixture that a household cook would purchase and then carry to a nearby small-scale commercial mill to create a unique *laxoox*/*canjeero* flour blend

**Fig. 4:**
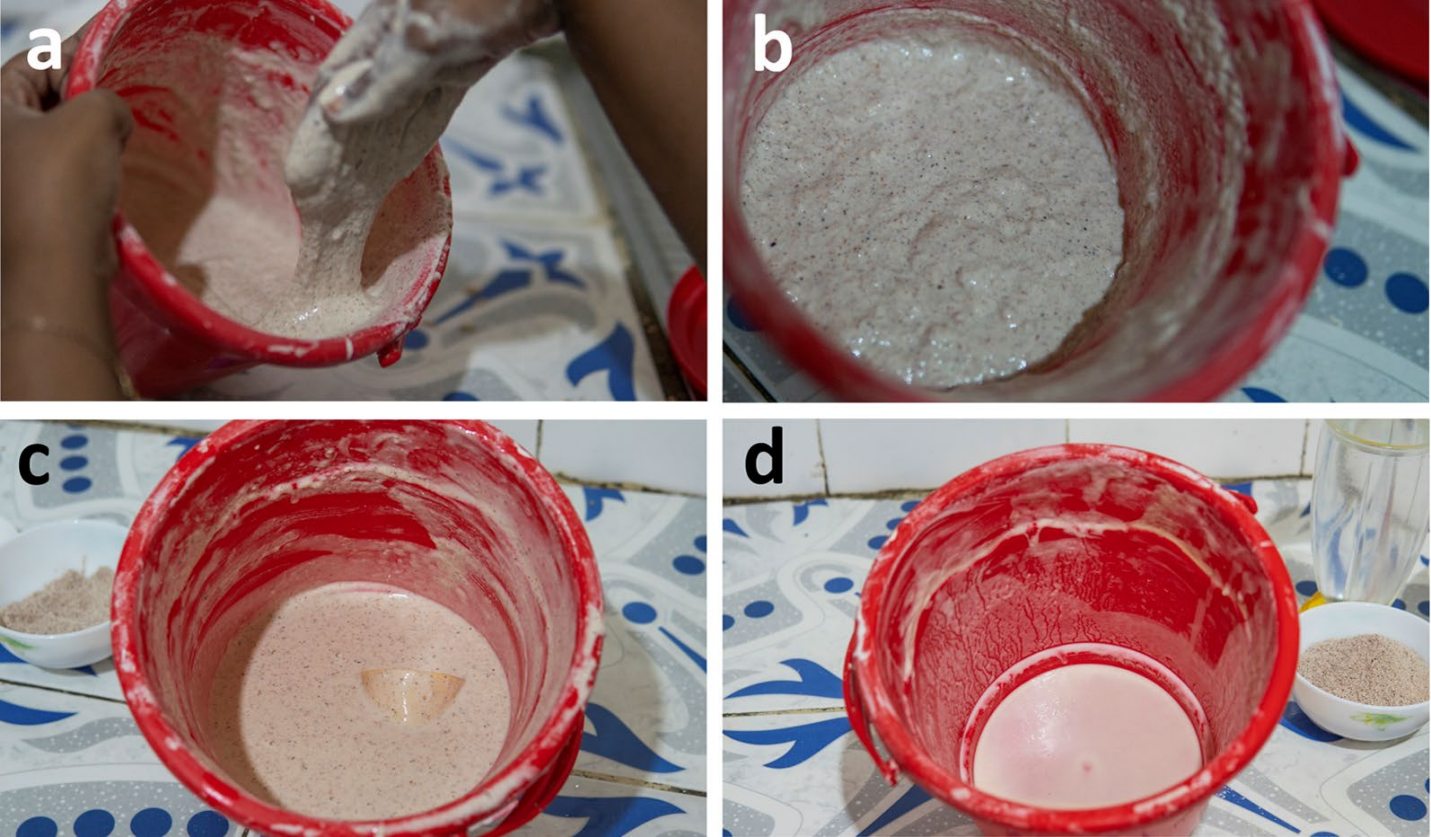
Dough preparation steps. **a** Mixing batter by hand in Hargeisa (a new heritage zone) inside a plastic container, the most common material for mixing and fermenting flatbread batter. **b** The flatbread batter halfway through mixing as it becomes increasingly aerated and bubbly in a process that takes 10–15 min. **c** The flatbread batter after mixing, typically bubbly, smooth, and velvety in texture. Photo credit: Mustafa Said. **d** Fermented *dhanaanis* (starter) reserved from a flatbread batter in Hargeisa along with batter residue on the interior surface. This container will be used, unwashed, for subsequent batches to catalyze fermentation, a common method for ensuring adequate fermentation or in colder seasons, depending on the geographic location and corresponding climate. Photo credit: Mustafa Said

**Fig. 5:**
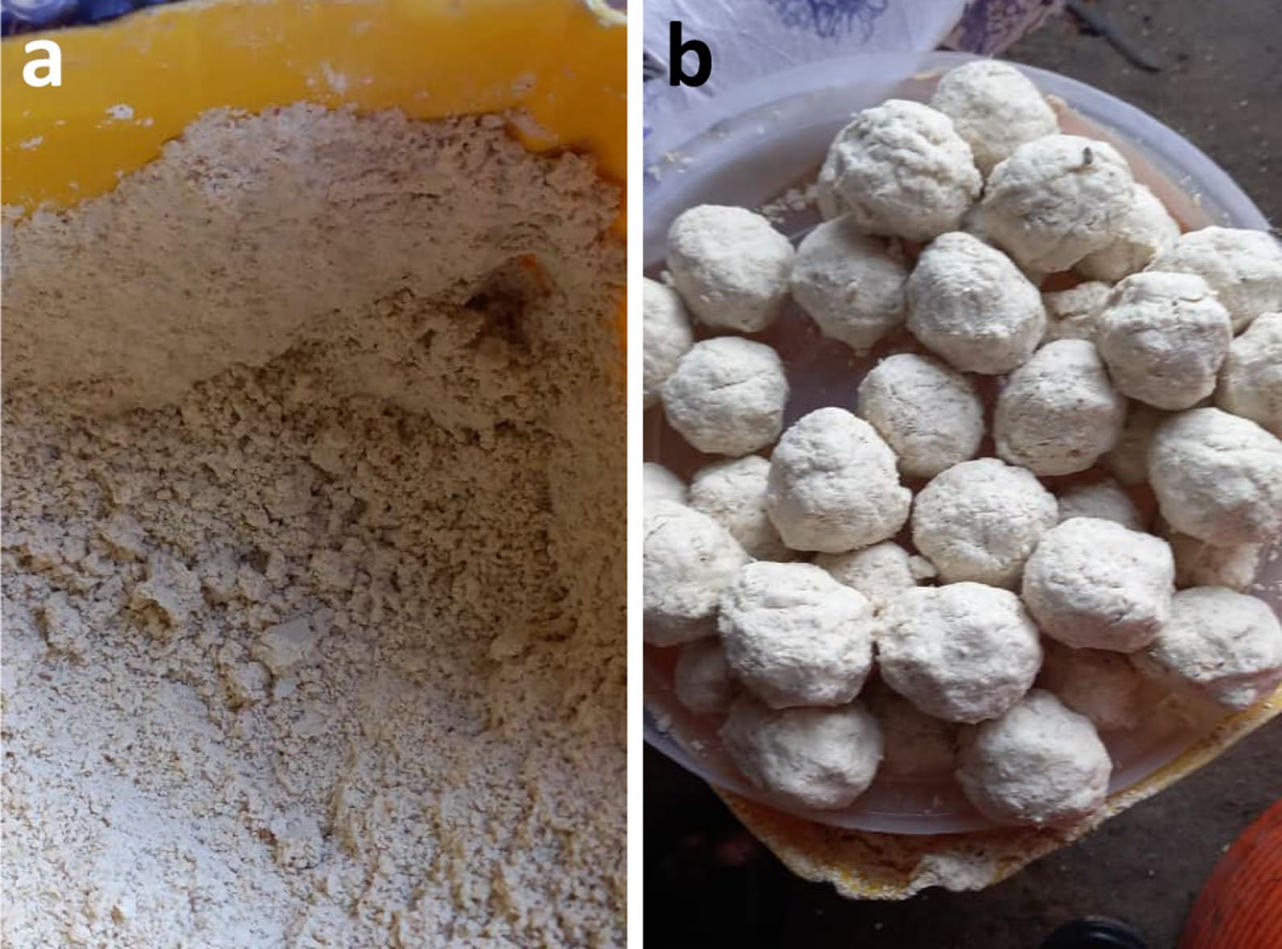
Commercially produced *cajiin* in Mogadishu, commonly a mixture of refined wheat and maize moistened with water. **a** Daily each morning, retail saleswomen provide *cajiin* producers with unique mixtures of grains and cereals to be processed into *cajiin* dough, and then retrieve their doughs, like this one, in the afternoon. **b** In the evenings, saleswomen prepare single-batch balls of *cajiin* for sale in local markets to customers who use the *cajiin* to prepare their flatbread batter at night, allow it to ferment overnight, and cook it the following morning for breakfast. Photo credit: Ali Hussein

**Fig. 6:**
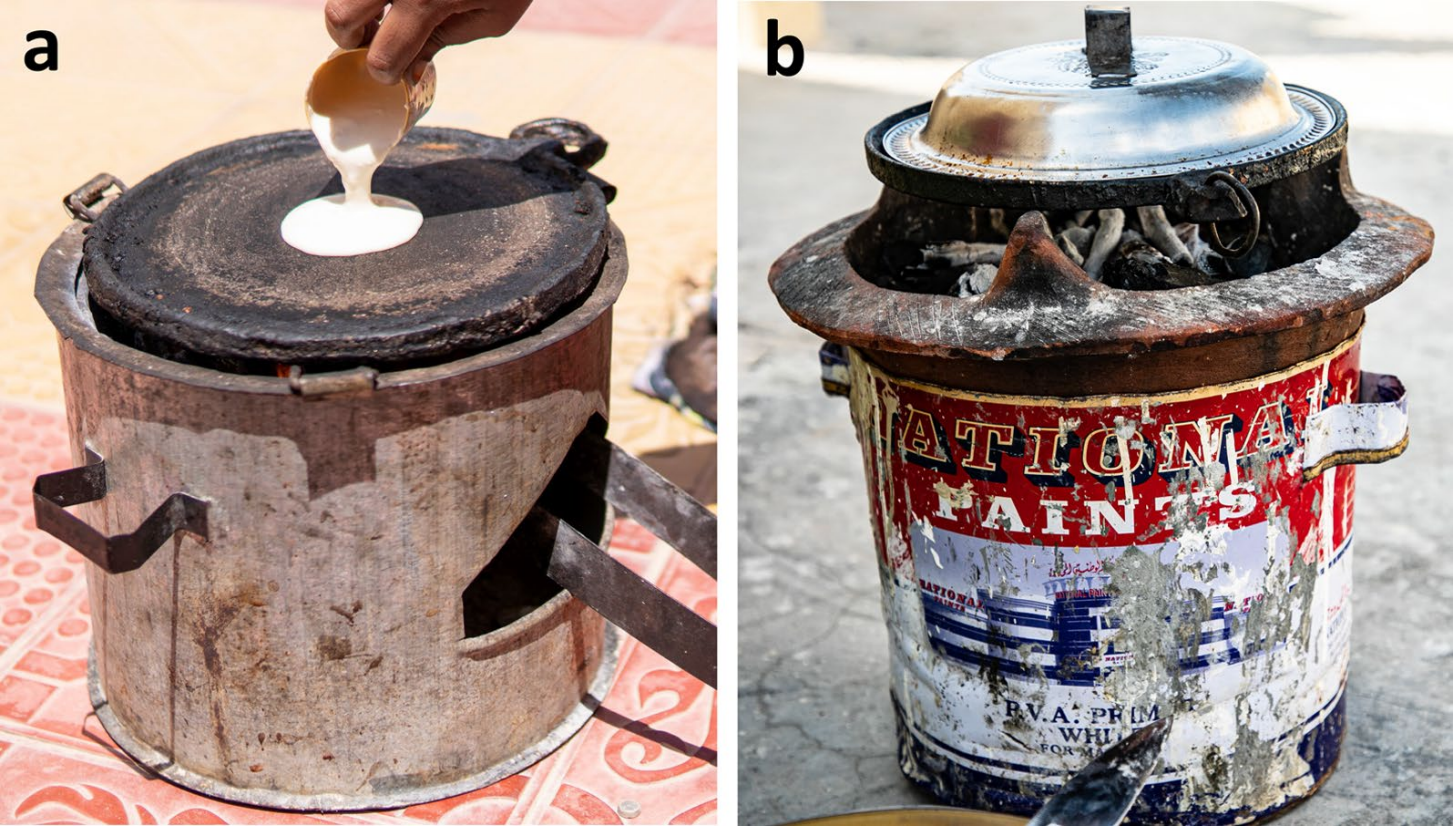
Fireboxes to bake *laxoox*/*canjeero*. **a** Firebox known as *girgire* in Hargeisa. **b** Firebox known as *burjiko* in Mogadishu. Fireboxes hold hot charcoals that heat the cast iron griddles from underneath. Before the advent of fireboxes, *laxoox/canjeero* was commonly cooked over wood fires on the ground. Today, the flatbread is also frequently cooked over gas stoves, depending on household means, cooking tools, and preferences. Photo credits: Mustafa Said (**a**) and Abdikarim Omar (**b**)

**Fig. 7:**
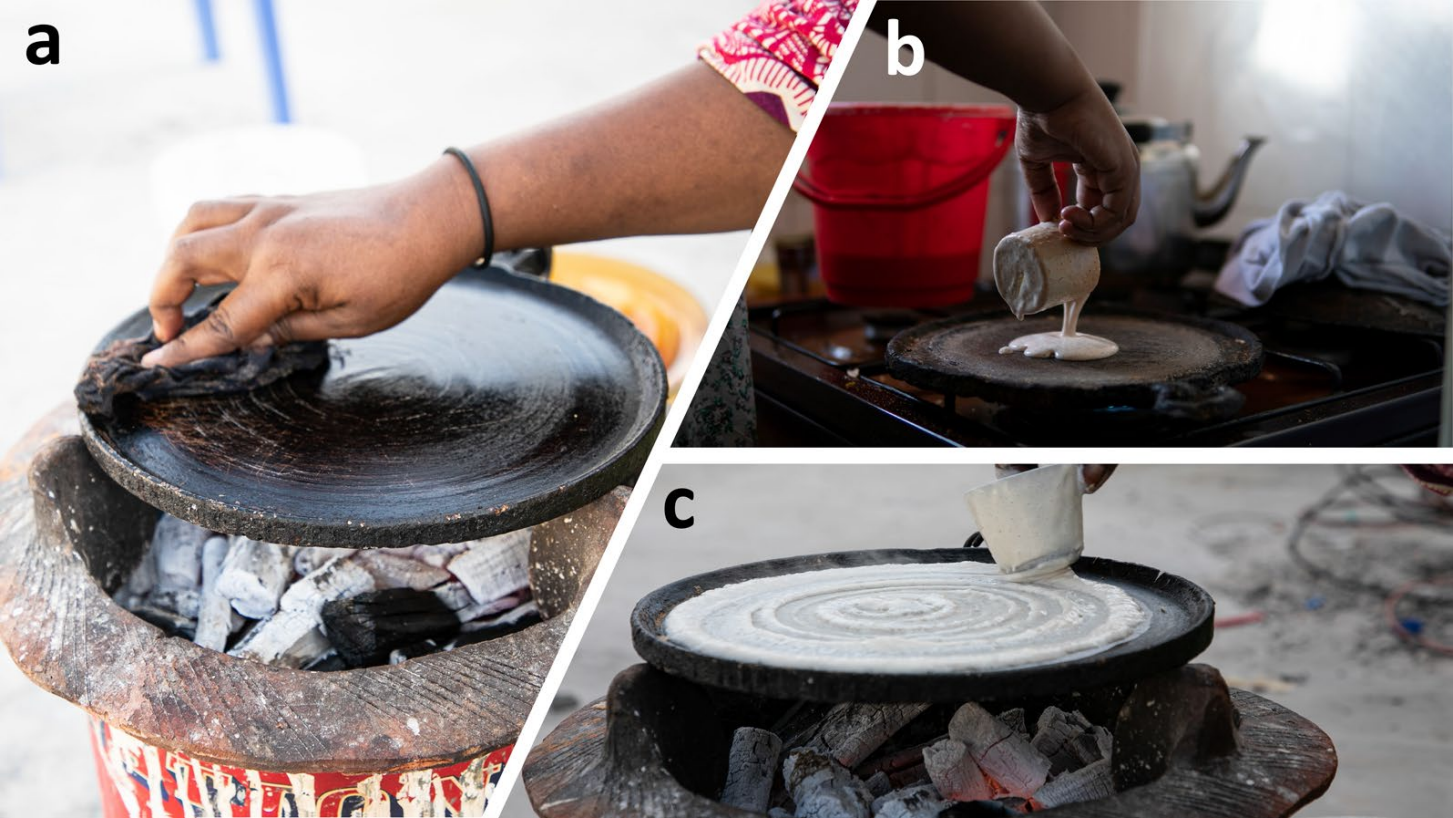
Shaping and baking *laxoox*/*canjeero*. **a** Preparing the griddle with vegetable oil using a blackened rag, known as a *masaxaad*, in Mogadishu (innovative zone). Alternately, the first cooked piece of *laxoox/canjeero* is folded and used to oil the griddle. In lieu of vegetable oil, some households use goat ghee to prevent the flatbread from sticking. Photo credit: Abdikarim Omar. **b** Pouring the batter on a cast iron griddle atop a gas stove in Hargeisa (new heritage zone). Anecdotes from interviewees indicate that ceramic griddles pre-date cast iron griddles in Somalia and may have been crafted and sold by tradespeople from the Arabian Peninsula. Today, the cast iron griddle is ubiquitous for this purpose in research locations, while online sources show that other griddle types, including non-stick pans, are used in global production. Photo credit: Mustafa Said. **c** Shaping the batter on the griddle over a firebox (*burjiko*) in Mogadishu (innovative zone) using a flat-bottomed cup. In a circular motion, the cook pushes batter outwards from the center of the griddle, creating a spiralized effect. The cup never touches the surface of the griddle but leaves behind it a thin trail of batter, while the un-flattened batter puffs up around it. The shape and patterns of the finished flatbread are a signal of quality. Photo credit: Abdikarim Omar

**Fig. 8:**
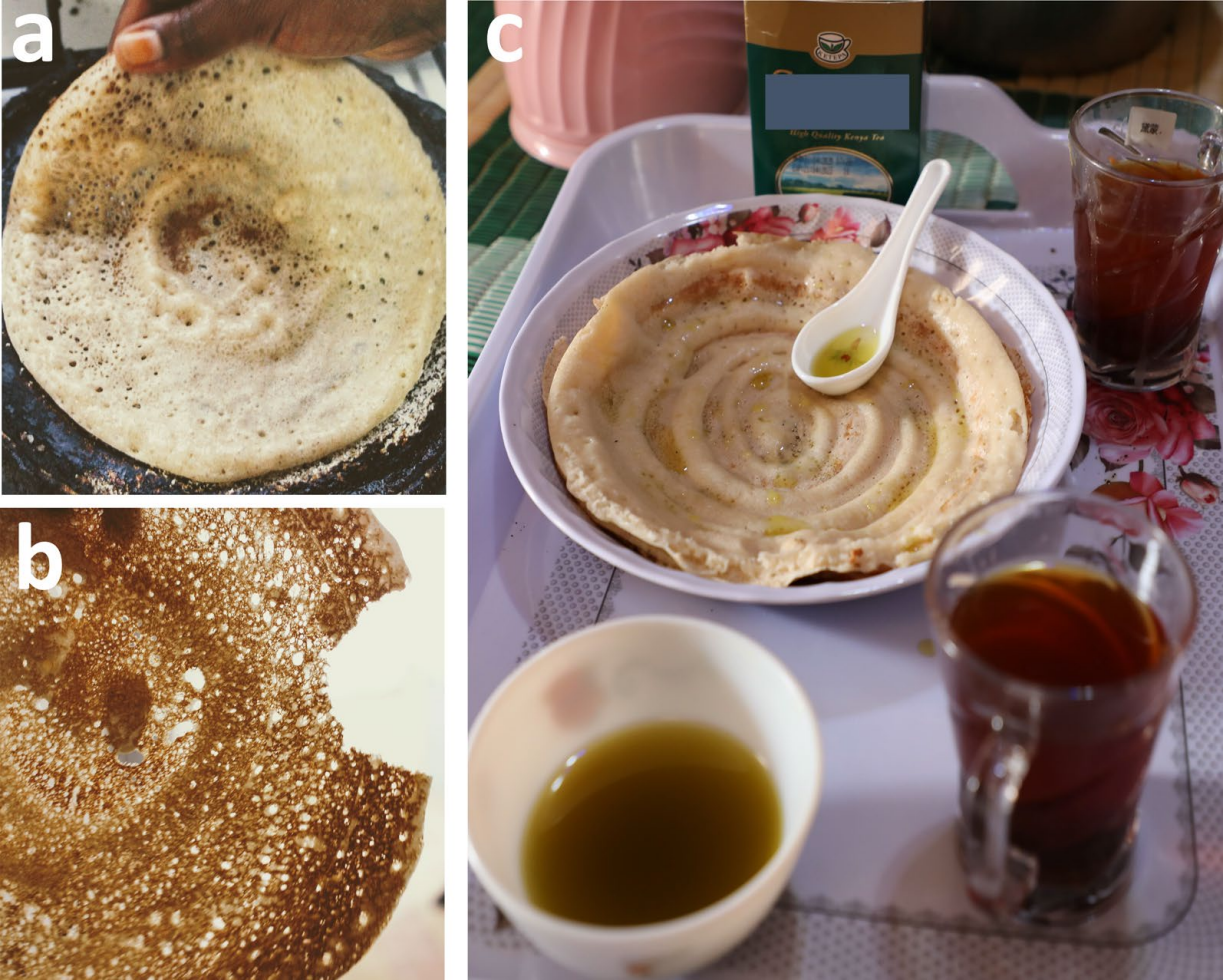
*Laxoox*/*canjeero* appearance and consumption. **a** Cooked *laxoox/canjeero* retains a soft, puffed side that never contacts the griddle surface but rather rises via steam under a well-fitting lid; and a browned side that cooks on the oiled griddle and is crispy when just cooked but quickly softens. The flatbread is pockmarked with holes, or “eyes,” and appears translucent when held up to a light source. Photo credit: Erin Wolgamuth. **b** A plate of flatbread served for breakfast in Mogadishu (innovative zone), sprinkled with sugar and accompanied by oil and tea. All respondents reported eating this flatbread for breakfast with either sweet (tea, sugar) or savory (meat or vegetable) dishes. Some also consume *laxoox/canjeero* at other meals and with other dishes. Photo credit: Abdikarim Omar

**Fig. 9:**
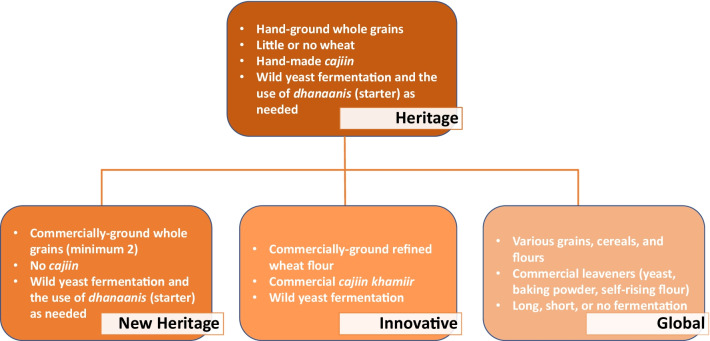
Production styles flowchart, from heritage to new heritage, innovative and global. The latter three groups developed concurrently and were not chronologically subsequent. Heritage production is linked to the historic era of seminomadic Somali pastoralists, while differences among the subsequent three production styles have resulted from the Somali civil conflict and continue today

The respondent data enabled the identification of discrete *laxoox/canjeero* processing steps, which are illustrated in Fig. 2. Data on each step (batter ingredients and preparation, *cajiin* preparation, leavening, shaping, and cooking), as well as consumption patterns, are shown in Figs. 3, 4, 5, 6, 7, 8, and 9 and Tables 1, 2, 3, and 4 and discussed in the following sections.

**Table 1:** Specific processing characteristics of *laxoox*/*canjeero* production related to the preparation of batter

	Respondents (%)
Additional ingredient (besides flour and water)*	
Salt	56
Sugar	12
Eggs	7
Spices	40
*Cajiin*	37
*Geographical location of cajiin users*	
Southern Somalia	100
Somaliland and Ethiopia	0
*Geographical location of users of refined wheat flour only*	
Southern Somalia	100
Somaliland and Ethiopia	0
*Water temperature*	
Regular (room temperature)	77
Warm	23
*Material of mixing container*	
Plastic	91
Wood or metal	9
*Color of batter after mixing*	
White color	50
Brown color	36
Yellow color	14
*Aeration*	
With bubbles	67
Without bubbles	33
*Batter consistency*	
Thin consistency	53
Thick consistency	47

*More than one answer admitted

**Table 2:** Specific processing characteristics of *laxoox*/*canjeero* production related to batter leavening

	Respondents (%)
*Leavening agent*	
Spontaneous fermentation	77
Addition of starter (*dhanaanis*)	23
*Fermentation location*	
Kitchen	86
Cool area (unspecified)	7
Inside the home (separate from kitchen)	5
In a sunny location outdoors (daytime fermentation)	2
*Fermentation length*	
Overnight	100
Indicators of fermentation readiness*	
Presence of bubbles	100
Sour odor	49
Formation of a clear layer of liquid on top of batter (ethanol)	9
Increased batter volume	5
*Responses to appearance of ethanol*	
Mix ethanol into batter	79
Pour off ethanol and cook underlying batter	19
Discard batter	2
Hot season changes*	
Reduced starter amount	36
No changes	34
Shorter fermentation time	30
Addition of cool water	2
Cold season changes*	
Longer fermentation time	65
Increased starter amount	47
Addition of commercial yeast	26
Keeping the container unwashed	23
Adjusting fermentation temperature (close to a lighted fire or charcoal)	14

*More than one answer admitted

**Table 3:** Specific processing characteristics of *laxoox*/*canjeero* production related to baking

	Respondents (%)
*Baking system*	
Stainless steel griddle pan with lid	100
Other systems	0
Griddle pan preparation for reducing sticking*	
Greasing with oil or goat ghee at the beginning	100
Greasing between each bread	53
Sprinkling large salt flakes	42
Heat source*	
Somali firebox (*girgire*)	77
Gas stove	21
Wood fire on the ground	7
*Heat intensity*	
Medium	95
High	5
Low	0
*Baking time*	
2–5 min	100
Indicators of completed cooking*	
Light browning of bread color on underside	47
Sound of steam escaping from the lid edges	35
Steam visible formation	19
Baked odor	12
Presence of “eyes” or holes in the bread surface	7
Time estimate based on experience	7
*Days elapsed before consumption, after baking*	
Less than 1 day	42
1 day	33
2 days	21
3 days	4

*More than one answer admitted

**Table 4:** Consumption patterns of *laxoox*/*canjeero*

	Respondents (%)
Meal*	
For breakfast	100
For lunch (in Ethiopia only)	26
For dinner (in Ethiopia only)	36
For *iftar* (event meal to break the daily fast during Ramadan)	19
Accompanying foods, dishes, or beverages*	
Tea	86
Diced, sauteed meat and vegetables (*suqaar*)	70
Meat stew (*maraq*)	51
Sauteed goat or lamb liver (*beer*)	49
Eggs (boiled, fried, or scrambled with vegetables)	42
Goat ghee (*subag*)	40
Sauteed vegetables (*dalac bilash*)	30
Oil (commonly sesame or other vegetable oil)	21
Minced, sundried meat preserved in ghee (*muqmad* or *oodkac*)	16
Honey (*malab)*	14
Sugar	12
Goat or lamb kidney (*kalyo*)	9
Sauce (e.g., tomato)	7
Beans	7
Coffee (*qaxwo*)	5
Lentils (*misir*)	5
Stewed fava beans (*foul medames*)	5
Milk	2
Other goat meat	2
Fish soup	2
Spinach	2

*More than one answer admitted

### Batter ingredients and preparation

Across research locations, 95% of respondents used wheat flour in their flatbread batter, which ensures gluten development to achieve a soft and chewy texture. Seventy percent used more than one grain, cereal, or grass in the batter, while 30% used only wheat flour (soft wheat, *Triticum aestivum* L. or, occasionally, durum wheat, *Triticum durum* Desf.). Refined wheat flour, known as *daqiiq* in Somaliland and *bur* in Somalia (see glossary of terms - Additional file 3), was the most common wheat flour used. Respondents also used flours such as sorghum (*Sorghum bicolor*) (55%), locally known as *hadhuudh* or *masago*, while some mentioned a white, early maturing variety known locally as *hadhuudh yar yare*. Other flours used in this flatbread are maize (*Zea mays* subsp. *mays*) (32%), including a yellow variety known as *galay*, and a white variety known as *galayda cad*; barley (*Hordeum vulgare* L.) (18%), known as *heedh*; teff [*Eragrostis tef* (Zucc.) Trotter] (7%), noted only among respondents in Ethiopia’s Somali State; and cowpea [*Vigna unguiculata* (L.) Walp] (7%), known as *digir* (Fig. 3).

Respondents indicated that *laxoox*/*canjeero* was historically composed of non-glutinous flours (namely sorghum) at least as far back as the 1960s. Conversations with Somali elders recorded in Mogadishu in the 1980s show that sorghum and maize were used in that era in southern Somalia (Lower Shabelle and Brava) [14]. Given that *laxoox/canjeero* is a griddle-cooked flatbread, this research supports the suggestion that nonindigenous cereals, like wheat, were adopted in Somalia (as in Ethiopia and Yemen) after local grains and baking technologies were established [15]. This research also supports the assertion that griddles are linked to the use of indigenous African grain varieties and are archaeological indicators of the culinary use and consumption of those plants [15].

Today, whole grains, cereals, and grasses are sold in bulk markets or packaged in retail shops (Fig. 3). Respondents who purchased whole grains in the market regularly made use of mechanical mills for hire, typically situated nearby. In all cases, respondents easily acquired milled wheat, grains, and cereals.

Additional dry ingredients (Table 1) included salt (used by 55% of respondents), sugar (11%), and other flavorings (39%) like garlic, fenugreek (*Trigonella foenum-graecum* L.), and black cumin (*Nigella sativa* L.) seed. Three respondents added egg to their batters; all reported that this improves the appearance of the flatbread. Egg yolk proteins are characterized by emulsifying properties which improve gas retention in batters [21], leading in this case to a puffier *laxoox/canjeero*.

Seventy-six percent of respondents used room-temperature tap water to hydrate the batter, while the remainder, all of whom live in climates with variable temperatures that include cold seasons, used warm water (Table 1).

Most respondents (91%) prepared the batter in a plastic container (Fig. 4). Exceptions include traditional wood or stainless steel containers (Table 1). Respondents described a range of qualities for their pre-fermentation batters. Batter colors ranged from white and yellow to red and brown, corresponding to the use of whole or refined flours. Some mixed batters (56%) showed bubbling and varying degrees of viscosity (47% reported a “thin” versus thick consistency). Additional descriptions from respondents indicated that the bubbles fade and the batter becomes “less sticky” and “soft like gum” during the vigorous hand-mixing process which may last 10–15 min and yields a very smooth, velvety texture.

### *Cajiin* preparation

Historically, gluten-like structure development in *laxoox/canjeero* relied on *cajiin* (Fig. 5), a pre-gelatinized dough made from sorghum (and/or other non-glutinous or low-gluten grains) and hot water. *Cajiin* was produced in a lengthy manual process requiring 1 to 2 days of intermittent activity. At some point in the late twentieth century, industrial-grade kneading machines were introduced in various cities including Mogadishu, Hargeisa, Burao, Baidoa, and Warsheikh, to produce commercial quantities of *cajiin* dough. This greatly reduced the labor-intensiveness of the production process for household cooks; however, only a handful of machines remain; those in Somaliland cities were destroyed or dismantled during conflict leading up to Somalia’s civil war and never repaired or replaced. At the time of the survey, 100% of southern Somalia respondents used *cajiin* to prepare the flatbread, whereas in Somaliland and the Somali State of Ethiopia, none of the respondents used *cajiin* (Table 1).

*Cajiin* was historically produced by soaking a dough in just-boiling water and then kneading the water into the dough to create a slack batter. The treatment of flour with hot water changes protein and starch properties. (In some cases, it also decreases naturally occurring mycotoxins, resulting in a safer food product.) More specifically, a hydrothermal treatment causes starch to gelatinize, conferring hydrocolloid properties which, in bread, mimic gluten. Gelatinized starch provides the batter with gas-holding capacity because of increased viscosity [22], improving the stability of the dough and the structure and flexibility of the resulting bread. Thus, the use of *cajiin* is fundamental to achieving the desired texture in *laxoox/canjeero* made from low-gluten or gluten-free flours. It is less important in batters which include wheat flour, and unnecessary (although its use was observed in several cases) in batters made only with wheat flour.

Soaking *cajiin* in hot water resembles the preparation of *absit*, a fundamental ingredient in the production of *injera,* the prominent flatbread in neighboring Ethiopia [19] which is composed primarily of *teff* flour, a non-glutinous grain. Research shows that *absit* has a significant influence on the physicochemical and sensory qualities of *injera* [23–27]. Like *cajiin*, *absit* confers the desired texture and consistency of bread; without it, *injera* made of non-glutinous flours tends to be powdery and does not show a spongy structure with characteristic holes, known as “eyes” [28]. A similar comparison can be made between *cajiin* and *sharaba*, a slurry of flour and hot water used in the preparation of a fermented flatbread known as *lahoh* in nearby Yemen. Interestingly, hydrothermal treatment of flour, namely flour scalding, is popular also in eastern and Baltic countries, where it is frequently used to make heavily malted brown bread from rye flour. This treatment increases the porosity of the bread, deepens the color, and delays the staling process, all of which are also desirable characteristics of *laxoox/canjeero* [29].

Handmade production of *cajiin* has fallen out of favor, while the availability of glutinous wheat flours has increased in recent decades, including in rural areas. In Somaliland and in Ethiopia’s Somali State, where there is no mechanized *cajiin* production, the use of *cajiin* in flatbread production has ceased entirely. Only in urban, mainly southern Somali locations, has the use of *cajiin* continued, although it has been produced since circa the 1970s by industrial kneading machines.

The dry ingredient composition of *cajiin* has also changed over time. Whereas it was historically composed of sorghum, respondents in Mogadishu reported that it is currently made of maize, wheat, or sorghum flours, and sometimes flavored according to customer preferences. One *cajiin* producer noted that a simple maize dough is his most popular in terms of sales volume.

The mechanized process of commercial *cajiin* production is as follows: Each morning, retail *cajiin* saleswomen supply a mixture of whole dry ingredients (between 3 and 10 kg according to respondents) to *cajiin* producers, some of whom also own milling machines. The producer moistens the grains with room-temperature water and mills them by machine to a fine flour which is sieved to eliminate most of the bran. Then, hot water is added to the flour along with any flavorings, like salt or garlic, and the mixture is transferred to a sheeting machine where it is pressed between two large rollers and then manually gathered, folded, and reinserted between the rollers repeatedly to knead the dough. This continues for 25 to 60 min until a homogenous dough is formed. Saleswomen collect and pay for their doughs mid-afternoon and divide them into single-batch, ball-shaped portions (Fig. 5) for retail sale in local markets during evening hours. At night, the household cook adds the purchased *cajiin* to her flatbread batter to ferment until morning.

### Leavening

As in bygone eras, long fermentation persists in research locations. Most (77%) household cooks achieved leavening via spontaneous fermentation, relying on environmental wild yeasts (Table 2). Twenty-three percent of respondents enhanced fermentation with the addition of *dhanaanis* (Fig. 4), a microbial starter consisting of a portion of the previous day’s fermented batter. As a ratio example, one respondent used 120 g of *dhanaanis* to 750 g of flour. Depending on climate and seasonal conditions, respondents utilized either spontaneous or enhanced fermentation with facility. Among those relying on spontaneous fermentation, 70% used *dhanaanis* as a secondary (additional) fermentation agent if needed, mainly to mitigate the effects on fermentation of colder seasonal temperatures. Some respondents (23%) simply refrained from washing the fermentation container, thereby leaving behind a residue of fermented batter that, similarly to *dhanaanis*, speeds fermentation of a new batter. In warmer climates or seasons, respondents may wash the container daily to avoid over-fermentation or a perceived overly sour taste. Per interviews, this flatbread was historically fermented in the same manner. None of the respondents regularly used commercial yeast (Additional file 3).

*Dhanaanis* is a sour batter, similar to a sourdough (“mother culture”) but more hydrated. *Dhanaanis* is likewise comparable to Ethiopian *ersho*, a portion of batter from a previous batch used as starter in the preparation of *injera* [19]. Sourdough acts as a microbial starter, composed of yeasts and lactic acid bacteria (LAB), that is cyclically renewed by means of a back-slopping procedure [30], i.e., the introduction of a small amount of starter into fresh ingredients for further fermentation. The acidifying effect of *dhanaanis* is due to the production of organic acids (lactic and acetic) by LAB during leavening. The presence of organic acids also benefits bread shelf life by reducing mold growth during storage [31]. LAB have a healthy probiotic effect among consumers and their fermentation is increasingly appreciated in western countries.

The characteristic sour taste of sourdough bread is related to its low pH [32]. Although yeasts have the primary leavening role, producing carbon dioxide and ethanol, LAB release important flavor-responsible volatile compounds, such as alcohols, aldehydes, ketones, and esters [29]. Flavor varies according to the microbial composition of sourdough and the type of cereal/flour used, as well as environmental conditions. Despite an appreciation of the health benefits of fermentation, most consumers, across all cultures, prefer sweet and salty foods and dislike sour and bitter ones [33–35]. Western consumers generally expect milder sour taste, including in sourdough bread [36]. Likewise, research respondents expressed a preference for a less pungent sour odor and taste. In Somaliland and Ethiopia, the *dhanaanis* (starter) yields an increasingly sour flavor with each batch; once a household’s maximum tolerance for sour flavor is reached, all of the batter is cooked or discarded (no *dhanaanis* is reserved), and the household cook begins the batter-making process anew. In areas where batters are *cajiin*-based, household cooks generally use wild yeast fermentation and do not reserve any *dhanaanis* because of its perceived overly sour taste, although they may do so during colder seasons to enhance fermentation.

As the kitchen is usually the warmest room in the home, 86% of respondents fermented the batter there overnight. In urban locations such as those surveyed for this research, the kitchen is often a separate or semi-exposed structure outside the main residence and therefore may retain a different ambient temperature compared to the home; 5% of respondents fermented their batters “in the home” as opposed to in the kitchen. Only 7% of respondents fermented the batter in an unspecified “cool area;” all lived in the relatively hot, coastal city of Berbera. For overnight fermentation, the containers are usually covered to keep out animals and debris, and sometimes wrapped in a towel or cloth to retain heat and encourage fermentation. Finally, 2% of respondents prepared flatbread during the daytime for evening consumption and fermented the batter outdoors in a sunny location.

In addition to manipulating the type or quantity of fermentation agent in response to seasonal temperature changes, respondents also made adjustments to the fermentation period (length of time) and location. More respondents adjusted the fermentation period in cold seasons (65% fermented the batter for more time) than in hot seasons (30% fermented the batter for less time), perhaps indicating that mitigating cold temperatures is more of a challenge than mitigating hot temperatures. In cold seasons, respondents took the following additional measures: increased the fermentation temperature by placing the batter near a lighted fire or charcoal (14%), used more starter (47%), or added commercial yeast (26%).

Before cooking the batter, respondents observed various indicators of readiness, the most common of which (100%) is the presence of bubbles at the surface. Additional indicators were a sour smell (49%) and, less commonly, increased batter volume (5%) or the occasional formation of a clear liquid layer on the surface of batter (presumably ethanol derived from fermentation) (9%). Interestingly, respondents differed in their perceptions of the cause of liquid formation. Some believed that it indicates over-fermentation, while others believed the opposite. In the context of household cooking, which is totally reliant on environmental conditions without effective temperature controls and without the use of commercial starters, the composition and behavior of lactic acid bacteria and yeasts can vary significantly. When yeasts are more abundant or more active than LAB, they may produce greater amounts of ethanol during fermentation [30], which is clearly perceivable in the form of a distinct liquid layer.

Respondents, who were not aware of the exact nature of the liquid, differed in their reactions to it: 79% mixed the liquid into the batter, waited a short period for additional bubbling on the surface of the batter, and then proceeded to cook the flatbread. Others poured off the liquid and cooked the remaining batter, while 2% discarded the entire batter, believing both the liquid and the corresponding batter to be unhealthy. Among respondents who mixed the liquid into the batter, 29% also added flour to maintain the desired consistency, while 18% gently heated the batter by adding warm water or tea, or by placing it in the sun for a short time. Six percent, believing that the liquid indicates underfermentation, added commercial yeast to the batter and waited a short time before proceeding to cook.

Higher amounts of ethanol decrease the extensibility and strength of gluten-containing dough and can alter the physical properties of a non-gluten batter [37]. In this case, respondents who add flour to the batter and then gently heat it are most likely to counteract the effects of higher levels of ethanol that would significantly change the texture or taste of the flatbread. The variety of documented responses to liquid formation may demonstrate that knowledge and skills about batter development have not kept pace with the introduction of wheat flour (gluten) to an historically non-glutinous or low-gluten bread.

Finally, several respondents also confused the purposes of *dhanaanis* and *cajiin*. In terms of food technology, *cajiin* and *dhanaanis* play different roles, the first being a structuring agent, and the second a fermentation starter with acidifying properties. In Warsheikh, where a *cajiin* machine recently came into disrepair, one respondent said “Nowadays I use *dhanaanis* because the [*cajiin*] machine is not working. I reserve [fermented batter or *dhanaanis*] in a separate container because I do not have [*cajiin*] dough… but we are expecting to repair our machine soon *Insha’Allah*.” Others used *dhanaanis* or commercial yeast to supplement or replace *cajiin* depending on seasonal temperature changes. Still others insisted that making the flatbread with *cajiin* is preferable to making it with *dhanaanis*, because *cajiin* creates better fermentation and a superior taste and smell, or because of a perception that use of a fresh dough is healthier than a fermented starter. These answers indicate that, over time, household cooks may be losing traditional bread-making knowledge. The existing literature on other cultures and communities likewise documents a trans-generational loss of knowledge about traditional and indigenous food production, including flatbread [38].

### Shaping and cooking

Many home kitchens are powered by charcoal stoves of different types, including in urban areas. These stoves include built-ins replete with chimneys and multiple “burners” as well as the *girgire* (known in southern Somalia as a *burjiko*), which is a light, portable, metal firebox (Fig. 6). Among respondents, 77% used wood charcoal to cook flatbread, while others used gas stoves (21%) or wood fires (7%) (Table 3). Ninety-five percent of respondents used medium-strength heat to warm the cooking pan and throughout the entire cooking process.

*Laxoox* and *canjeero* are usually prepared on a flat cast iron griddle with short sides, called a *dhaawe* or *dawa* [14], set above the heat source. Griddles are appropriate for non-gluten-producing breads and were suitable to Somalis’ historically nomadic lifestyle, as opposed to ovens. Ceramic griddles in neighboring Ethiopia date as far back as 520 BCE, while dome-shaped clay griddle lids, used today in metal form to make *laxoox*, appear later in the early second millennium CE [39]. Whereas bread ovens (named *foorno* in southern Somalia and *moofo* in the north) are only found where Near Eastern cereals (especially wheat) are used in bread-making, the use of griddles corresponds archaeobotanically to the use of non-glutinous grains [15]. Other ancient baking systems such as the *tannur* (Arabic, pl. *tananir*) [15], known as *tinaar* (alt. spelling *tinnaar*, pl. *tinaarro*) in Somalia, where they are used in southern zones [14], are not appropriate for baking *laxoox*/*canjeero*, because non-glutinous grains do not provide the necessary dough consistency required for baking on the vertical walls of these kinds of clay ovens.

All respondents prepared the griddle with oil (usually imported sunflower or another vegetable oil) or ghee made locally from goat milk, to prevent the *laxoox*/*canjeero* from sticking (Fig. 7). They spread the oil or ghee on the pan using a blackened cloth known as a *masaxaad* or using a folded piece of flatbread, usually the first cooked piece. Some respondents (53%) reapplied oil to the griddle as needed during the cooking process. Some also rubbed large flakes of salt on the pan before cooking to further reduce sticking (42%).

Using a flat-bottomed cup, which serves as both ladle and tool to spread the batter, the cook scoops batter from the fermentation container and pours it onto the center of the griddle (Fig. 7). Working quickly, she gently places the cup at the center of the pan, touching the batter but not pressing through it, and makes a spiral pattern, moving outwards and spreading the batter to cover the pan to its perimeter (Fig. 7). The trail left behind the cup is a thinner, almost translucent portion of the batter, while the rest is thicker and opaque; this creates an attractive spiral effect.

The bread is quickly covered by a domed lid that allows the batter to puff while trapping condensation to keep it from drying out. The high moisture level of the batter and the use of a lid serve to steam-leaven the bread and contribute to rapid starch gelatinization that traps gas bubbles and produces an open, spongy structure with pock marks which, similarly to Ethiopian *injera*, are known as “eyes” [40]. Lifting the lid, the cook will quickly check the underside of the batter for a golden-brown shade, and then—often with a butter knife—she gently lifts the cooked bread off the pan and onto a plate. Only one side of the bread directly touches the pan and becomes crisp; the other side, especially the thicker portion of the spiral, is cooked through (without flipping) but very soft. This spiral pattern (Fig. 8) is appreciated as a sign of talented cookery and is apparently distinctive among regional flatbreads. While batter for Ethiopian *injera* is applied to the griddle in the same manner, the spiral disappears as the batter spreads and is not a signature of the cooked flatbread. Though a thorough comparison has not been undertaken, this difference in appearance may be the result of a higher degree of viscosity in *injera* as compared to Somali *laxoox/canjeero*. Other differences between these fermented flatbreads include size (*injera* is typically much larger, cited at 60 cm in diameter on average [19] compared to the average 15–19 cm of *laxoox/canjeero*) and sour taste (*injera* batter is usually fermented for several days before cooking [19], whereas *laxoox/canjeero* batter is fermented for one night thus it has a less intense sourness).

Respondents heeded various signals to know when the bread is finished cooking: light browning of the underside of the flatbread (47%), the hissing sound of condensation dropping from the interior of the lid onto the hot pan (35%), visible steam formation (19%), the smell of cooked batter (12%), or a simple estimate of elapsed time (7%) after many years of daily production. Cooking time ranged between two and five minutes (respondents were not asked to time this, only to estimate).

Once baked, the flatbread is quickly consumed: 42% of respondents reported to consume it immediately, 33% kept it for 1 day, 21% for 2 days, and 4% for 3 days.

### Consumption patterns

*Laxoox*/*canjeero* is traditionally served at breakfast; indeed, 100% of respondents reported eating it for breakfast compared to only 26% at lunchtime and 36% at dinnertime (Table 4). In comparison, 100% of respondents in Ethiopia’s Somali State consider *laxoox*/*canjeero* to be suitable for lunch or dinner in addition to breakfast. Furthermore, 19% of respondents eat *laxoox*/*canjeero* at *iftar*, the sunset meal eaten by Muslims to break the day’s fast during the holy month of Ramadan, a finding which corresponds to observations reported elsewhere [12].

Across research locations, respondents reported eating this flatbread with similar dishes, including with oil, sugar, and tea (Fig. 8) or with savory accompaniments. The most common (86%) accompaniment to flatbread was sweet Somali tea, followed by diced and sauteed meat with vegetables (*suqaar*, 70%), meat stew (*maraq*, 51%), and sauteed goat or lamb liver (*beer*, 49%). Following in popularity were eggs (42%, either boiled, fried, or scrambled with vegetables), ghee (40%, typically goat ghee, known as *subag*), and an economical composition of sauteed vegetables known as *dalac bilash* (30%). Twenty-one percent of respondents reported eating *laxoox/canjeero* with oil (typically sesame or another vegetable oil combined with sugar and eaten with tea), or with grilled goat or lamb kidney (*kalyo*, 9%), while 16% eat it with minced, sundried meat preserved in ghee known as *muqmad* in Somaliland and northern areas, and as *oodkac* in southern areas. Fourteen percent of respondents reported consuming *laxoox/canjeero* with honey, while a few (7%) eat it with beans, tomato sauce (7%), coffee (*qaxwo*, 5%), lentils (*misir*, 5%), and the Arab dish of stewed fava beans known as *foul medames* (5%) which originated in Egypt and is very common in nearby countries, namely Ethiopia, Eritrea, and Sudan, under the name of *shahan ful* [41]*.* Finally, respondents also mentioned eating *laxoox/*canjeero with other goat meat dishes (2%), fish soup (2%), spinach (2%), and milk (2%). No pork-based dishes were reported, as pork is prohibited (*haram*) in Islam [42], the prevailing religion among Somalis.

These results demonstrate the very wide range of foods consumed alongside this bread, which is regularly present at Somali meals, especially breakfast.

### The historical evolution of *laxoox/canjeero:* an original framework of production styles

While *laxoox*/*canjeero* production was found to be relatively homogenous across locations and among respondents, the data revealed two significant divergences: (1) in the procedure adopted for structure development, and (2) in bread formulation. With regard to the first divergence, historical production of *cajiin* appears to have been adapted in two distinct manners since circa the 1970s, the era during which respondents recall a shift from long-standing hand production to mechanization of certain steps. In southern Somalia, especially in urban and peri-urban zones, the use of *cajiin* has continued but is no longer handmade; rather, it is produced by industrial machines and sold commercially in local markets. In contrast, respondents in Somaliland and Ethiopia’s Somali State did not report the use of *cajiin* in their flatbread batter. Per interviews, this difference is borne of the period of conflict preceding the Somali civil war which led to discrepancies in access to commercial *cajiin* kneading machines across Somalia and Somaliland.

The other significant divergence revealed by the data pertains to the formulation of bread, in particular the types of flour. While 30% of respondents used only wheat flour (whole or refined), 70% combined more than one grain or cereal in a batter mixture almost always (95%) including wheat flour. The divergence appears when comparing geographic zones: among respondents in southern Somalia, 68% made *canjeero* composed solely of wheat flour, while 32% used a mixture of grains and cereals. In stark contrast, 100% of respondents in Somaliland and Ethiopia’s Somali State used at least two grains or cereals in their batter and in some cases as many as six or seven, in a dry mixture known as *budo*. These findings on divergences indicate some degree of transformation in the production of *laxoox/canjeero*, especially during the most recent half-century. This contrasts existing works which find that Ethiopian *injera* preparation has preserved traditional practices for centuries, if not millennia [19], a relatively rare scenario in light of globalization, and evidenced primarily in rural areas per the existing literature [43, 44].

The nuances of production shown in the data led to the development of an original framework which categorizes the differences in four styles: one is historical (“heritage”), while the other three are current (“new heritage,” “innovative,” and “global”) (Fig. 9). These original naming conventions, discussed below, are used for the first time in this article.

### Heritage production: the importance of *cajiin*

Heritage production sheds light on the ways in which flatbread production styles have evolved differently across Somali regions, going back at least as far as the memories of living respondents and continuing up to the 1970s. The methods discussed here are informed by oral histories and by the memories of interview respondents and are not exhaustive.

Historically, *laxoox/canjeero* was composed of the most commonly available whole grains, especially sorghum, a native African crop which was domesticated on the northeastern savannas of Sudan sometime before 2000 BCE [45]. Heritage production was a two- to three-day intermittent process involving a variety of steps and tools. Since *laxoox*/*canjeero* was a breakfast staple, production was initiated daily, even in pastoralist and agro-pastoralist households, evidence that cereal processing and bread baking technologies are intricately linked activities of rural life [15].

Heritage production required processing by hand of whole grains that may have been grown locally (as in the case of sorghum and maize in the early twentieth century) or imported (wheat as well as sorghum and maize). Over time, imported wheat flour was incorporated where available for purchase. All steps of flatbread preparation, including the processing of whole grains, were undertaken by women. This supports cross-cultural ethnographic studies [46, 47] which show that in non-mechanized societies like Somalia, the final stages of agricultural processing such as pounding, sieving, hand-sorting, and grinding, tend to fall in the domain of women.

**Fig. 10:**
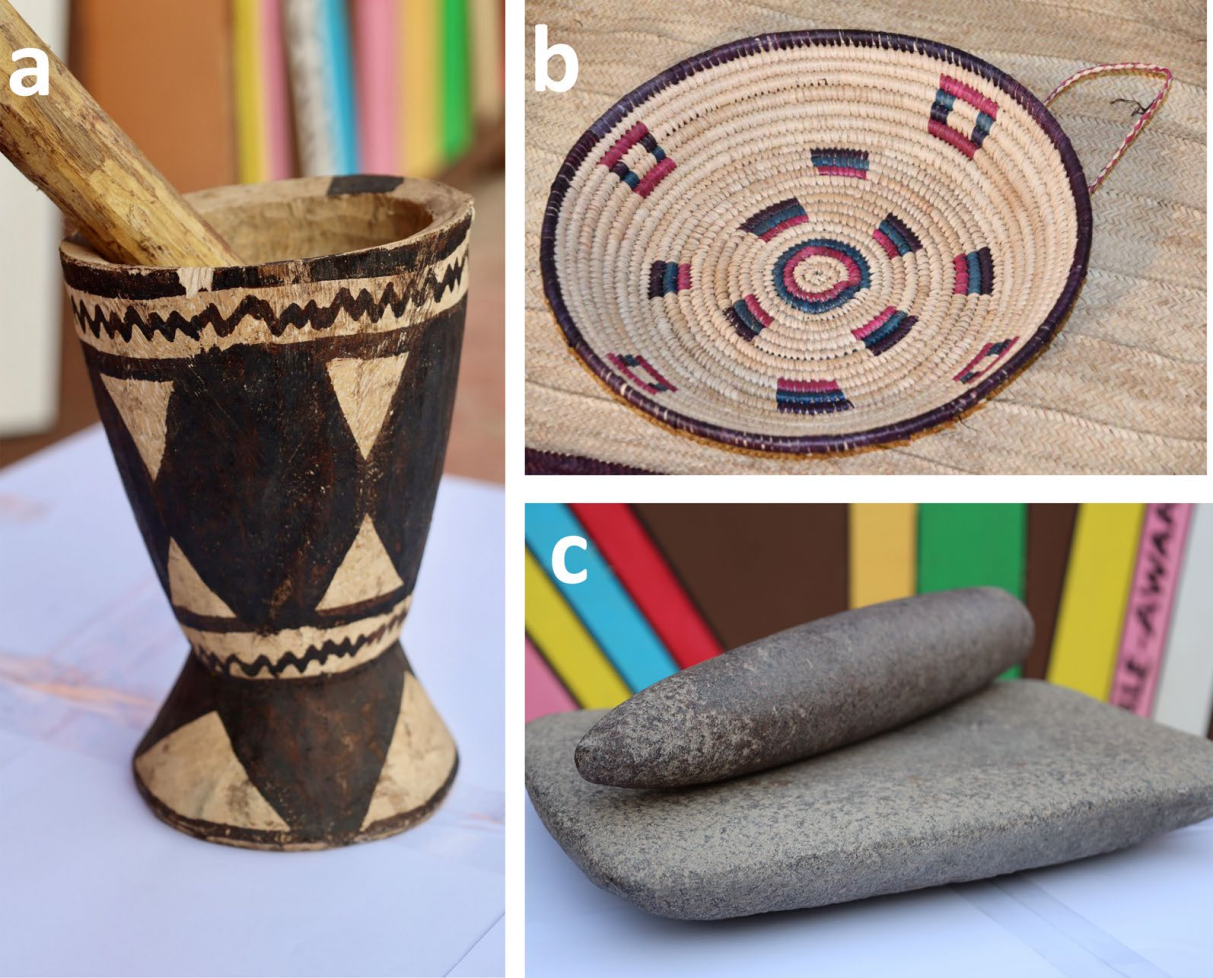
Traditional tools used in Somali flatbread preparation. **a** A mortar and pestle used to crush whole grains. **b** A traditional *masaf* to winnow grains, traditionally woven from local plants. **c** A *mixdiin* and a *caali*, made from locally procured stones and used to crush grains and cereals or work fine doughs. Photos (**a**, **c**) courtesy of the Hargeysa Cultural Centre in Somaliland, photo credit Jean Omboke. Photo (**b**) courtesy of the Somali Museum of Minnesota, USA

The historic predominance of low-gluten and non-glutinous flours meant that the use of *cajiin* was essential to achieving the preferred structure and texture of *laxoox/cajiin.* Household cooks manually developed the *cajiin* through a process of pounding the moistened grain with a pestle and mortar (*tib* and *mooye*) (Fig. 10) to separate grain from chaff, then winnowing the grain in a large, shallow, woven basket called a *masaf* (Fig. 10)*,* while removing debris. The winnowed grain was soaked in water and then dried on a cloth in direct sunlight for up to 1 hour. These steps were sometimes repeated to ensure clean and malleable grains.

The winnowed grains were then mixed with a small amount of water and processed by hand using a saddle quern-stone called a *mixdiin* (a relatively flat surface stone) and *caali* (a rolling pin-like stone held in the hand and used to crush grain mixtures against the stone) [14] (Fig. 10) for around 1 hour to form a very soft *cajiin* dough. Every community had at least one saddle quern, but not every household, so they were often shared among community members. The *cajiin* was formed into a ball and placed in a bowl with very hot or boiling water. Once cool enough to touch, the sorghum dough, water, and a small amount of wheat flour (if available) were vigorously kneaded by hand for as long as 15 min before the batter was set in a warm area (inside the home or near a fire) to ferment overnight. The next morning, the batter was cooked on a griddle.

At the time of cooking, women reserved *dhanaanis* to activate fermentation of that evening’s batter for cooking the following morning. Somali territories include varied climates; deep knowledge about the effects on the batter of weather, temperature, and time enabled women to determine how much *dhanaanis* would achieve ideal fermentation.

Respondents recount that industrial-style kneading machines arrived in major cities in Somalia in the 1970s and 1980s. The machines were literally life-changing for household cooks who had access to them and could assemble their preferred mixture of *cajiin* ingredients at home, bring it to machine operators in the market, and have it mechanically processed for a small fee; hours of labor became a matter of standing in a queue. In events leading up to the start of the Somali civil war in 1991 (which saw Somaliland’s assertion of independence), Somaliland cities and territories were bombed, looted, and much infrastructure was destroyed [48]. According to interviews, looters disassembled and made off with building materials and machine parts, including from businesses. *Cajiin* kneading machines in the capital city of Hargeisa and the commercial center of Burao were among those either destroyed or stolen and have not been replaced. Indeed, further disparities between economic development in the north and in the south, and between the food economy zones, i.e., agro-pastoralists, pastoralists, riverine, and urban communities, have been reported [49]. No other machines in Somaliland have been identified in our research; thus, if any were functional prior to the war, it is possible that they were likewise destroyed.

In urban locations in southern Somalia, the kneading machines are still used today. Essentially everywhere else in Somalia, Somaliland, and Ethiopia’s Somali State, the use of *cajiin* has ceased. Not only *is cajiin* production labor intensive, but imported refined wheat flour is nearly ubiquitous nowadays, rendering unnecessary the gluten-like structure development of heritage-style flatbread.

### Contemporary bread production

In an original categorization of contemporary *laxoox/canjeero* production, we have identified three styles that resulted from the Somali civil conflict and continue today: new heritage production, innovative production, and global production. New heritage and innovative production correspond broadly to geographic zones and are shown in the map in Fig. 1.

- **New heritage production** is found in Somaliland, in Ethiopia’s Somali State, and in rural areas of southern Somalia without access to *cajiin* kneading machines. It preserves the use of whole grains and cereals, typically in mixtures of at least two varieties (i.e., *budo*), although household cooks in these areas consistently include refined wheat flour in the flatbread batter. While some rely solely on wild yeasts to ferment the batter, new heritage production has largely continued the use of *dhanaanis* to enhance batter fermentation.
- **Innovative production** is found namely in urban southern and eastern Somalia where commercial kneading machines are available. While this style continues the long-standing use of *cajiin*, household cooks rely on modern technology (industrial-grade kneading machines) and commercial production to obtain it. Notably, those who use *cajiin* are knowledgeable and capable of using *dhanaanis* if circumstances require it, for example if cold weather slows down fermentation or if the *cajiin* machines are temporarily unavailable, as was the case in Warsheikh at the time of data collection. Respondents purchase a small quantity of *cajiin* each day to create a fresh batter. The same respondents who use *cajiin* have largely forgone the use of varied whole grains and cereals in the batter, with an overall reliance on refined wheat flour which has become more available in Somalia since the 1970s and 1980s, and the consumption of which has been promoted worldwide via westernization [15].
- **Global production**, while not a focus of this research, is worth mentioning because it also resulted from civil conflict, specifically from population displacement. The Somali diaspora is significant; between 1990 and 2015, the number of people born in Somalia but living outside it more than doubled [50], and displacement continues due to climate-driven threats [51]. Somali household cooks around the world have made use of locally available ingredients and products, such as commercial yeast [52], baking soda or powder [53], pancake mix [54], and kitchen blenders, to create this traditional flatbread with new techniques. Some of these techniques have also been adopted in Somalia and Somaliland, where access and knowledge allow. For example, one respondent who grew up in rural northern Somalia recalled making daily *laxoox* for her family as a child using skillful manipulation of wild yeasts and homemade starters, but today uses a blender in her Mogadishu home to quickly mix an unfermented batter comprised largely of oat flour and with the use of baking soda.

## Conclusions

The results of this in-field survey have shown that *laxoox*/*canjeero* production is relatively homogenous, but with two significant divergences: in bread formulation and in the procedure adopted for bread structure development, due to disparities in the mechanization of production between rural and urban or peri-urban areas, and between areas differently impacted by the civil war. Due to security challenges, ongoing conflict, and the presence of SARS-COVID-19 during research, it was not possible for data collectors to access a comprehensive breadth of locations to conduct interviews. Nevertheless, given the relative homogeneity of production methods for *laxoox*/*canjeero*, the data presented here offer an adequate picture and a strong foundation for further research. Further analysis of historical geo-culinary trajectories and linguistic data, local archaeobotany, historical trade, agriculture, and consumption patterns will contribute to deeper understanding of this flatbread and its evolution. Better understanding of its origins would help shed light on the reasons behind certain production methods for this flatbread and others in the region, especially *injera* in Ethiopia and *lahoh* in Yemen.

## Supplementary Information

**Additional file 1.** Questionnaire on *laxoox/canjeero*.

**Additional file 2.** Questionnaire on *cajiin*.

**Additional file 3.** Glossary of terms.

## Data Availability

All data and materials have been presented in the paper.
