# Supplementary material for: A survey of laxoox/canjeero, a traditional Somali flatbread: production styles
Source: J. Ethn. Food. 2022 Jun 21;9(1):22. doi: 10.1186/s42779-022-00138-3 (PMC9210053; doi:10.1186/s42779-022-00138-3)
Supplement: Supplementary file 1 — Additional file 1. Questionnaire on laxoox/canjeero. [file 42779_2022_138_MOESM1_ESM.docx]

**Supplementary Material 1. Questionnaire on *Laxoox / Canjeero production***

**Questions for:**

1) Adult women who regularly prepare *canjeero* for their household (or used to prepare it in the past)

2) Can be family (mothers, sisters, wives, aunts, etc.), friends, neighbors, or other “convenient respondents”

3) Each respondent must be from a different household and different “household of origin.” For example:

- **Do not** interview 2 sisters who learned to make *canjeero* from the **same** person (mother). Only interview one of them.
- **Do not** interview your mother and your mother’s sister/*habo* who learned to make *canjeero* from the **same** person. Only interview one of them.
- **You can** interview your mother and your father’s sister/*eedo* who learned to make *canjeero* from **different** people

4) Residence versus origin: Respondents **do not** have to be originally from the place they live now, but must be originally from southern Somalia (or “*canjeero* area”)

5) Adapt the flatbread naming in the questionnaire using the appropriate local name. In locations where people eat *canjeero* (i.e., Baidoa, Warsheikh, Mogadishu, Marka, and Garowe), respondents have to be asked about *canjeero*, whereas the word *laxoox* has to be used in Hargeisa, Berbera, Beer, and Jigjiga.

Interview date: _______________________ Interview place: ________________________

Age:______ Place of birth/origin:_____________

*Researcher Note: The questions below are very specific, with the intention of understanding the food science behind canjeero preparation. Please feel free to let the conversation flow naturally, but take notes and follow up as needed to get answers to each question.*

**Part I: Because you prepare *canjeero* regularly for your household, I would like to ask you a few questions about how you prepare it. These are detailed questions so that someone who has never seen or eaten *canjeero* can understand how you prepare it.**

1. How do you make the *canjeero* *qosh*? (*Qosh* = batter)
   1. At what time do you usually prepare the *canjeero* *qosh*?
      1. Hour: _____________ **or**
      2. Salat (prayer): _____________
   2. With which flours, like *bur* (refined wheat flour) and grains like *masago* (sorghum), *qamadi* (whole wheat), any others, do you usually make *canjeero*? List all with af Somali names:
      1. ___________________________
      2. ___________________________
      3. ___________________________
      4. ___________________________
      5. ___________________________
      6. ___________________________
   3. Are they whole (brown/red) grains and flours or refined (white) grains and flours?
      1. Whole grains __________
      2. Refined _____________
      3. Mix _____________
      4. Other ________________________
   4. Do you buy the grains and flours already milled **OR** do you buy them whole and then take them to a mill machine?
      1. Buy already milled/ ii *shiid* from the shop _________
      2. Buy whole grains and take to the mill machine _____________
      3. Other ____________________________
   5. When you prepare the *qosh*, what are the amounts of each flour and grain that you use?
   6. Do you add warm water or regular temperature water to the *qosh*?
      1. Warm water ____________
      2. Regular temperature _____________
      3. Other _____________________________
   7. Do you put any other spices or flavors in the *qosh*, for example garlic, *xawaash* (a mix of spices often including cinnamon, clove, cardamom, black pepper), *xulbad* (fenugreek), *geedo* (herbs), or others?
   8. What do you usually use to ferment (make *dhanaan* / *khamiir*) the *qosh*? For example *cajiin* *khamiir*, *dhanaanis*, or nothing (natural long fermentation), or other?
   9. Do you use any other method sometimes? What is it? Why?
   10. In what container do you prepare the *qosh*? What material (straw, plastic, wood, etc.)?
   11. With what tool do you mix the *qosh* (for example hand, wooden spoon, metal spoon, etc.)?
       1. Mix with hand ___________________
       2. Mix with spoon ___________________
       3. Other __________________________
   12. How does the *qosh* look after you mix it?

Color: _________________

Bubbles (Yes/No): __________

Consistency (Thin/thick): ______________

Other observations:

- 1. How does the weather (hot or cold) affect how you prepare *canjeero*? Does the weather affect how much *dhanaanis* you use? Does the weather change what time you prepare the *qosh* (earlier or later)?

1. How do you ferment the *qosh*?
   1. In what container does the *qosh* ferment (same as mixing container or different? If different, which material?)?
   2. Where does the *qosh* ferment (For example, in the kitchen? Is it a hot or cool area? How do you know where to put it?)
   3. For how much time does the *qosh* ferment? (For example, one night, or how many hours)
      1. How do you know the *qosh* is ready to cook?
      2. What color?: _______________
      3. What smell (sour)?: __________
      4. Bubbles (Yes/No): _________
      5. Consistency (thin/thick)_______
      6. Other observations:
   4. When it is time to cook the *canjeero*, if a liquid has formed on top of the *qosh*, what do you do with the liquid (for example, mix it into the *qosh*, pour it out, or something else)?
   5. At what time do you usually cook the *canjeero*?
      1. Hour: ______________ **or**
      2. Salat: _______________
   6. Do you add anything to the *qosh* before cooking?
   7. Do you mix the *qosh* again before cooking?
   8. Do you cook all of the *qosh* or save some (like *dhanaanis*)? What do you do with the reserved *qosh*? Where do you keep it?
2. How do you prepare the heat to cook?
   1. To cook the *canjeero*, where does the heat come from?
      1. Gas stove________
      2. Charcoal stove__________
      3. *Girgira* (traditional Somali firebox)____________
      4. Other ___________
   2. How is the heat for cooking *canjeero*?
      1. High/strong heat _________
      2. Low/weak heat __________
      3. Medium heat ____________
   3. What do you cook the *canjeero* on (for example, a pan)? What is the pan material?
   4. How do you prepare the pan? Apply oil? Apply salt?
3. How do you cook the *canjeero*?
   1. Do you cover the pan?
   2. Do you do anything to the pan between *canjeero*? For example, apply more oil, apply water, etc.
   3. How do you know when the *canjeero* is finished cooking?
4. After cooking
   1. At what time or meal do you usually eat *canjeero*?
      1. Morning/breakfast __________
      2. Midday/lunch _____________
      3. *Asriya*/snack _____________
      4. Dinner _____________
      5. Other______________
   2. With what foods or drinks do you take *canjeero*? (Describe **all for each meal**, feel free to use Somali names for dishes, like *suqaar*, *maraq*, etc.)
   3. For how long can you keep cooked *canjeero*?
   4. Do you wash the fermentation container or do you use it again the next day without washing?
   5. Are there any differences between the city (urban) and village (rural) preparation of *canjeero*? What are they?

      Probe: Especially fermentation and use of *cajiin khamiir*, or *dhanaanis*, or nothing (natural long fermentation).

**Thank you for your time!**
