# Supplementary material for: A survey of laxoox/canjeero, a traditional Somali flatbread: production styles
Source: J. Ethn. Food. 2022 Jun 21;9(1):22. doi: 10.1186/s42779-022-00138-3 (PMC9210053; doi:10.1186/s42779-022-00138-3)
Supplement: Supplementary file 2 — Additional file 2. Questionnaire on cajiin. [file 42779_2022_138_MOESM2_ESM.docx]

**Supplementary Material 2. Questionnaire on *cajiin*.**

**Questions for:**

1. Adult men and women who regularly prepare *cajiin* in Mogadishu for public purchase. This can be the people who operate the machinery, or the owners of the *cajiin* production facilities.
2. Preference for people who have done this job for some time, at least a few years.
3. Always preference for older people who can recall different ways of doing things from before. But not necessary.

Interview date: _______________________ Interview place: ________________________

Job (related to *cajiin*): ______________________

Age:______ Place of birth/origin:_____________

**Part I: Because you produce *cajiin*, I would like to ask you a few questions about it. These are detailed questions so that someone who is not familiar with *cajiin* can understand what it is.**

1. What is a simple explanation of *cajiin*?
   1. How is *cajiin khamiir* different from other types of *cajiin*?
2. Can you explain the process of producing *cajiin*?
   1. At what time do you usually prepare it? Is it prepared once per day or how many times per day?
   2. With which grains and flours do you usually make *cajiin*? List all with af Somali names:
      1. _______________________
      2. _______________________
      3. _______________________
      4. _______________________
      5. _______________________
      6. _______________________
   3. Are they whole grains and flours (usually red/brown) or refined (usually white/yellow)?
      1. Whole grains __________
      2. Refined _____________
      3. Mix _____________
      4. Other _______________
   4. What are the quantities of each ingredient you put into the *cajiin*? (For example kilos of corn, boxes of yeast, litres of water, etc.)
      1. _______________________
      2. _______________________
      3. _______________________
      4. _______________________
      5. _______________________
   5. Do you use warm water or regular temperature water to make the *cajiin*?
      1. Warm water ____________
      2. Regular temperature _____________
      3. Other ________________
   6. Do you put any other spices or flavors in the *cajiin* (for example salt, garlic, or *xawaash*, or other)?
   7. How do you use the mixing machine to mix the *cajiin*?
   8. For how long (how many minutes) does the *cajiin* get mixed by machine?
   9. How does the *cajiin* look after it goes through the mixing machine?
   10. How does the weather (hot or cold or rainy) change how you prepare *cajiin*?
3. After the mixing machine, where do you put the *cajiin*?
   1. Who divides the *cajiin* into small quantities for sale? (Is this the producer’s job or the saleswomen?)
4. Which is the most popular type of *cajiin* that sells the most on a daily basis? (*Cajiin khamiir* or a different *cajiin*)?
5. I understand people use *cajiin khamiir* to make *canjeero*. Do people use it to make anything else?
   1. What do people make with the other types of *cajiin* (not *khamiir*)?

**Part II: Now I would like to ask you a few general questions about *cajiin*.**

1. What are the advantages of using *cajiin* instead of *dhanaanis* or regular yeast to make *canjeero*?
2. When did people begin to use *cajiin* instead of *dhanaanis* to make *canjeero*? (What time period – 1970s, 1980s, 2000s, or other?)
3. From where came the idea to use *cajiin* to make *canjeero*? Who did this first?
4. Is it possible to make *cajiin khamiir* at home? Why do people prefer to buy it?
5. Does your facility make any other food products besides *cajiin*? What are they?
6. Bonus question: What are the differences between *canjeero* and *laxoox*?

**Thank you for your time !!**
