# Supplementary material for: A survey of laxoox/canjeero, a traditional Somali flatbread: production styles
Source: J. Ethn. Food. 2022 Jun 21;9(1):22. doi: 10.1186/s42779-022-00138-3 (PMC9210053; doi:10.1186/s42779-022-00138-3)
Supplement: Supplementary file 3 — Additional file 3. Glossary of terms. [file 42779_2022_138_MOESM3_ESM.docx]

## **Supplementary Material 3. Glossary of terms.**

| **Term** | **Meaning** |
| --- | --- |
| *Asriya* | Afternoon tea and snack coinciding with the afternoon (*asr*) prayer |
| *Beer* | Sauteed goat or lamb liver, eaten with *laxoox*/*canjeero* |
| *Budo* | A mix of grain and cereal flours unique to individual households or retail sales points |
| *Bur* | Refined (white) wheat flour (name used predominantly in southern Somalia) |
| *Burjiko* | Somali firebox known as *girgire* in northern areas |
| *Caali* | A baton-shaped tool made of stone and used to crush grains by hand against a flat *mixdiin* stone |
| *Cajiin* | A dough made historically of ground sorghum and water, and more recently of sorghum, maize, and/or whole wheat flours and water. |
| *Dalac bilash* | An economical dish of odds and ends served at home, e.g., sauteed vegetables, eaten with *laxoox*/*canjeero* |
| *Daqiiq* | Refined (white) wheat flour (name used predominantly in northern areas and Ethiopia’s Somali State) |
| *Dawa* or *dhaawe* | Cast iron griddle |
| *Dhanaanis* | Starter dough, reserved from fermented flatbread batter and used in the subsequent day’s batter |
| *Dhardhaar* | Historical heating source for cooking in rural locations composed of wood set atop three large stones, analogous to Ethiopian cooking technology |
| *Digir* | Cowpea |
| *Foul medames* | Stewed fava beans (Arabic name for this dish originating outside Somalia, namely in Egypt) |
| *Geedo* | Herbs |
| *Galay* | Maize (yellow) |
| *Galayda cad* | Maize (white) |
| *Girgire* | Somali firebox (known as *burjiko* in southern areas) |
| *Hadhuudh* | Sorghum (name used predominantly in Somaliland) |
| *Hadhuudh yar yare* | Sorghum (white, early maturing variety) |
| *Heedh* | Barley |
| *Iftar* | Sunset meal that breaks the day’s fast during the Muslim holy month of Ramadan |
| *Injera* | Ethiopian fermented flatbread bearing some resemblance in appearance and production to Somali *laxoox*/*canjeero* |
| *Kalyo* | Grilled goat or lamb kidney, eaten with *laxoox*/*canjeero* |
| *Khamiir* | Soured or fermented |
| *Laxoox* | Flatbread (name used predominantly in Somaliland and Ethiopia’s Somali State) |
| *Canjeero* | Flatbread (name used predominantly in southern Somalia) |
| *Malab* | Honey |
| *Maraq* | Meat stew |
| *Masago* | Sorghum (name used predominantly in southern Somalia) |
| *Masaxaad* | A black cloth used to oil the griddle before cooking the flatbread, and re-oil it as needed during the cooking process. |
| *Masaf* | Shallow basket used to winnow grains |
| *Misir* | Lentils |
| *Mixdiin* | A large flat stone used as a surface for crushing grains (paired with the *caali*) |
| *Mooye* | Mortar |
| *Muqmad* | Minced, sundried meat preserved in ghee, eaten with *laxoox*/*canjeero* (name used in Somaliland and northern Somali areas) |
| *Oodkac* | Minced, sundried meat preserved in ghee, eaten with *laxoox*/*canjeero* (name used in southern Somali areas) |
| *Qamadi* | Whole wheat |
| *Qaxwo* | Coffee |
| *Qosh* | Batter |
| *Rodhi moofo* | An oven-baked bread sold commercially in Somaliland |
| *Salat* | Prayer |
| *Subag* | Ghee (clarified butter), most commonly goat |
| *Suqaar* | Diced, sauteed meat with vegetables, eaten with *laxoox*/*canjeero* |
| *Tib* | Pestle |
| *Xawaash* | A mix of spices particular to the cook or shop, often including cinnamon, clove, cardamom, black pepper |
| *Xulbad* | Fenugreek |
